# Diverging regional inequalities in childhood diabetes, 1990–2023: a GBD 2023-based analysis

**DOI:** 10.48130/pedi-0026-0002

**Published:** 2026-06-11

**Authors:** Kexin Zhang, Chengxia Kan, Fang Han, Xuan Li, Tianpeng Zheng, Ningning Hou, Sufang Sheng, Wei Xu, Ying Xue, Xiaodong Sun

**Affiliations:** 1 Department of Endocrinology and Metabolism, Shandong Provincial Key Medical and Health Laboratory of Endocrinology and Metabolic Diseases, Affiliated Hospital of Shandong Second Medical University, Weifang 261031, China; 2 Clinical Research Center, Affiliated Hospital of Shandong Second Medical University, Weifang 261031, China; 3 Department of Endocrinology and Metabolism, Tongji Hospital, School of Medicine, Tongji University, Shanghai 200065, China; 4 Department of Physiology and Biophysics, University of Mississippi Medical Center, Jackson, MS 200065, USA; 5 Department of Endocrinology and Metabolism, The Second Affiliated Hospital of Guilin Medical University, Guilin 541199, China

**Keywords:** Childhood diabetes, Diabetes epidemiology, Inequality, Quality of care, Socioeconomic disparities, Machine learning

## Abstract

Childhood diabetes continues to rise, with wide variation across regions and income levels. We performed a cross-sectional analysis using Global Burden of Disease 2023 estimates for children aged 0–14 years across super regions, regions, and countries from 1990 to 2023. The primary objective was to characterize regional inequalities in incidence, mortality, and disability-adjusted life years, and to assess whether these inequalities are likely to persist in the future. Age-standardized incidence, mortality, and disability-adjusted life year rates were estimated per 100,000 population with 95% uncertainty intervals. Regional and national comparisons, temporal trend analyses, and socioeconomic gradient analyses were employed as core analyses. Risk factor attribution, the Quality of Care Index, Shapley additive explanations-based predictor contribution analysis, and Bayesian age-period-cohort forecasting were used as supportive analyses to interpret risk-attributable mortality patterns, care-related performance gaps, demographic and temporal contributors, and future incidence trajectories. From 1990 to 2023, childhood diabetes incidence increased in most regions, while mortality and disability declined overall. High-income North America had the highest incidence in 2023, whereas Western Sub-Saharan Africa had the greatest mortality and disability burden. Finland had the highest incidence, Qatar, Chile, and South Korea showed rapid incidence increases, and Nigeria had the highest mortality burden. Projections suggested stabilizing incidence in the United States but continued increases in China, India, Poland, Finland, and Qatar. Childhood diabetes shows persistent regional inequalities, highlighting the need for earlier diagnosis, reliable insulin access, and stronger pediatric diabetes systems in resource-limited settings.

## Introduction

Childhood diabetes is an increasingly prevalent chronic disease with lifelong consequences^[1,2]^. Early-onset diabetes exposes children to prolonged hyperglycemia, increasing the risk of cardiovascular, renal, and neurologic complications later in life^[3−6]^. Over recent decades, its epidemiology has evolved due to demographic transitions, environmental stressors, and expanded diagnostic capacity^[7]^. Understanding these transitions is essential to guide equitable prevention and care.

Our previous analyses using Global Burden of Disease (GBD) 2019 data revealed a persistent rise in childhood diabetes incidence from 1990 to 2019, alongside steady declines in mortality and disability-adjusted life years (DALYs)^[7]^. High-income regions exhibited the highest incidence, whereas low socio-demographic index (SDI) regions bore the heaviest mortality burden, underscoring deep inequities in pediatric care. The GBD 2021 update further identified that South Asia had the highest number of incident cases, whereas Eastern Sub-Saharan Africa remained the region with the highest mortality and DALY rates^[8]^. However, these aggregate estimates may obscure substantial intra-regional heterogeneity, where rapid diagnostic expansion, environmental exposures, and healthcare access interact in complex ways.

Since 2020, the COVID-19 pandemic has reshaped childhood diabetes patterns^[9]^. Viral triggers, lifestyle disruption, and psychosocial stress may have accelerated onset in predisposed children, while improved screening, insulin availability, and acute care helped lower mortality^[10−13]^. Together, these changes suggest that the post-pandemic era may represent a transitional phase in the epidemiologic trajectory of childhood diabetes^[13]^.

The updated GBD 2023 database incorporates expanded national registry data and enhanced regional modeling, improving estimate consistency and cross-country comparability^[14,15]^. The central question of this study was how regional inequalities in childhood diabetes burden changed from 1990 to 2023 and whether these inequalities may persist in the future. Therefore, we focused on temporal trends, regional and national differences, and socioeconomic gradients in incidence, mortality, and DALYs. Additional supportive analyses were used to contextualize risk-attributable mortality, care-related gaps, demographic and temporal contributors, and future incidence trajectories. This integrated framework aimed to clarify where childhood diabetes burden is increasing, where mortality and disability remain concentrated, and which care-related gaps may require greater attention.

## Methods

### Overview and data collection

We conducted a cross-sectional analysis using data from the GBD 2023 study, which provides internally consistent estimates of disease burden for GBD countries and territories from 1990 to 2023^[15]^. All analyses were conducted in accordance with relevant GBD data use and publication requirements. Childhood diabetes was defined as diabetes mellitus among individuals aged 0–14 years, based on GBD diagnostic criteria; in GBD 2023, total diabetes estimates for individuals younger than 15 years were based on the type 1 diabetes mellitus model. Data on incidence, mortality, and DALYs were derived from the *Global Health Data Exchange*. Estimates were generated using DisMod-MR 2.1, a Bayesian meta-regression model that synthesizes multiple data sources to ensure internal consistency across age, sex, location, and year. This study followed the STROBE guideline. Because only publicly available, de-identified, and aggregated GBD data were used, ethical approval and informed consent were not required.

The analytical framework was structured to distinguish core analyses from supportive analyses. Regional and national burden comparisons, temporal trend analyses, and socioeconomic gradient analyses were used to assess geographic heterogeneity, temporal changes, and development-related disparities in incidence, mortality, and DALYs. Risk factor attribution, Quality of Care Index (QCI) construction, Shapley additive explanations (SHAP)-based interpretation, and Bayesian age-period-cohort (BAPC) forecasting were used to contextualize risk-attributable mortality, care-related gaps, demographic and temporal contributors, and possible future incidence trajectories.

### Regional and national classification

Countries and territories were categorized according to the GBD 2023 super-regional and regional classification system to ensure consistency across geographic comparisons. Detailed region and country groupings are provided in Supplementary Method 1.

### Age-standardized rates

Age-standardized incidence rates, age-standardized mortality rates, and age-standardized DALY rates were expressed per 100,000 population using the GBD 2023 standard population. Age standardization was used to allow comparisons across locations and years independent of differences in population age structure. All estimates were reported with 95% uncertainty intervals (UIs).

### SDI-burden relationship analysis

The relationship between the SDI and the burden of childhood diabetes in 2023 was assessed across GBD regions. The SDI (range, 0−1) is a composite indicator reflecting income per capita, educational attainment, and fertility rate^[15]^. Scatterplots with LOESS curves and 95% confidence intervals were used to visualize associations between SDI and age-standardized incidence, mortality, and DALYs rates. Spearman correlation analysis assessed monotonic relationships.

### Joinpoint regression analysis

Joinpoint regression (Joinpoint Regression Program, version 5.0, National Cancer Institute, USA) was applied to assess temporal trends in ASR and detect significant inflection points. A log-linear model was fitted for each location and sex, with up to five joinpoints allowed and a minimum segment length of five years. For each segment, the annual percent change (APC) was estimated as (e^*β* − 1) × 100%, where *β* is the slope. The average annual percent change (AAPC) was calculated as the weighted mean of segment-specific APCs^[16,17]^.

### Mapping and EAPC calculation

Spatial patterns of change were visualized using choropleth maps of the estimated annual percentage change (EAPC) in ASRs from 1990 to 2023. EAPC was derived from the regression model ln(ASR) = *α* + *β* × year + *ε*, where EAPC = 100 × (e^*β* − 1). Each EAPC value was accompanied by a 95% CI derived from the standard error of the regression coefficient. Maps used color scales in which red indicates increasing and blue decreasing trends, ensuring color-blind accessibility^[18]^.

### Risk factor analysis

Risk-attributable mortality was assessed using the GBD 2023 comparative risk assessment framework. Population-attributable fractions and corresponding 95% UIs were derived from GBD-defined exposure distributions, theoretical minimum risk exposure levels, and relative risk functions. Regional and temporal variations in attributable mortality were summarized from 1990 to 2023.

### Calculation of the QCI

The QCI was constructed as a supportive analysis to provide a care-related interpretation of regional inequalities. Descriptive burden estimates identify where mortality and DALYs are concentrated but do not directly indicate whether these patterns are accompanied by weaker diagnosis, survival, or continuity of care. Therefore, QCI was used to integrate years of life lost to years lived with disability ratio (YLR), disability-adjusted life years to prevalence ratio (DPR), mortality-to-incidence ratio (MIR), and prevalence-to-incidence ratio (PIR) into a composite care-performance framework. Each indicator was standardized by z-transformation, and principal component analysis (PCA) was used to derive the composite score. Higher QCI values were interpreted as reflecting better care-related performance, including diagnosis, treatment, survival, and long-term management. Correlation and PCA analyses were used to assess the internal coherence and dimensional structure of QCI^[19]^. The added value of QCI was to assess whether regions with higher mortality and DALY burden also showed evidence of weaker care-related performance, rather than to define a separate disease burden endpoint Supplementary Method 2.

### HDI-EAPC relationship analysis

The association between socioeconomic development and temporal changes in childhood diabetes burden was further examined using the Human Development Index (HDI) and EAPC values. The HDI data were obtained from the United Nations Development Programme. Bubble plots visualized these relationships, with bubble size representing the number of cases in 2023. LOESS regression (95% CI) and Spearman correlation assessed nonlinear and monotonic associations.

### Machine-learning modelling

XGBoost models with SHAP interpretation were used as a supportive analysis to summarize demographic and temporal contributors to childhood diabetes burden in Western Sub-Saharan Africa and High-Income North America. Predictors included year, age group, sex, and log-transformed population size, and outcomes included incidence and DALY rates. Model performance was evaluated using ten-fold cross-validation, mean absolute error, and R^2^. SHAP values were used to summarize the relative contribution of each predictor to model predictions and to visualize nonlinear patterns^[20,21]^.

### Forecasting analysis

Future incidence trends were projected using a BAPC model that captures age, period, and cohort effects. The model employed a Poisson likelihood with log-linear predictors and second-order random-walk prior to ensure smooth temporal patterns. Posterior estimates were obtained through Integrated Nested Laplace Approximation (INLA), and model adequacy was evaluated using the Deviance Information Criterion and posterior predictive checks. Age-standardized incidence rates from 2024 to 2045 were forecast with 95% credible intervals^[22]^.

### Statistical analysis

All statistical analyses and visualizations were performed using R software (version 4.3.3; R Foundation for Statistical Computing). Age-standardized rates were used to quantify the burden of childhood diabetes across regions and selected countries.

## Results

### Temporal and regional dynamics of childhood diabetes burden

From 1990 to 2023, childhood diabetes incidence increased across most regions, while mortality and DALYs showed overall declining trends. High-income regions had the largest number of incident cases and rate. Compared with the 2021 GBD dataset, which identified South Asia as having the largest number of cases, the 2023 update identified High-Income North America as the region with the highest number of incident cases and the highest incidence rate, reporting 24,098 (11,817–49,989) new cases and an incidence rate of 36.54 (17.92–75.80) per 100,000 (Fig. 1; Supplementary Fig. S1 and Supplementary Table S1). Within this region, children aged 5–9 years contributed the largest proportion of new diagnoses, and a consistent male predominance was observed across all age groups (Supplementary Figs S2, S3). The most rapid increase in case numbers occurred in Sub-Saharan Africa (3.56-fold), particularly in Eastern Sub-Saharan Africa (5.10-fold), whereas Central Europe exhibited the fastest rise in ASIR (Supplementary Table S1). Spearman correlation analysis revealed a significant positive association between the SDI and incidence (r = 0.60, *p* < 0.001; *n* = 84), indicating that higher SDI values were linked to higher incidence rates (Supplementary Fig. S4).

**Figure 1:**
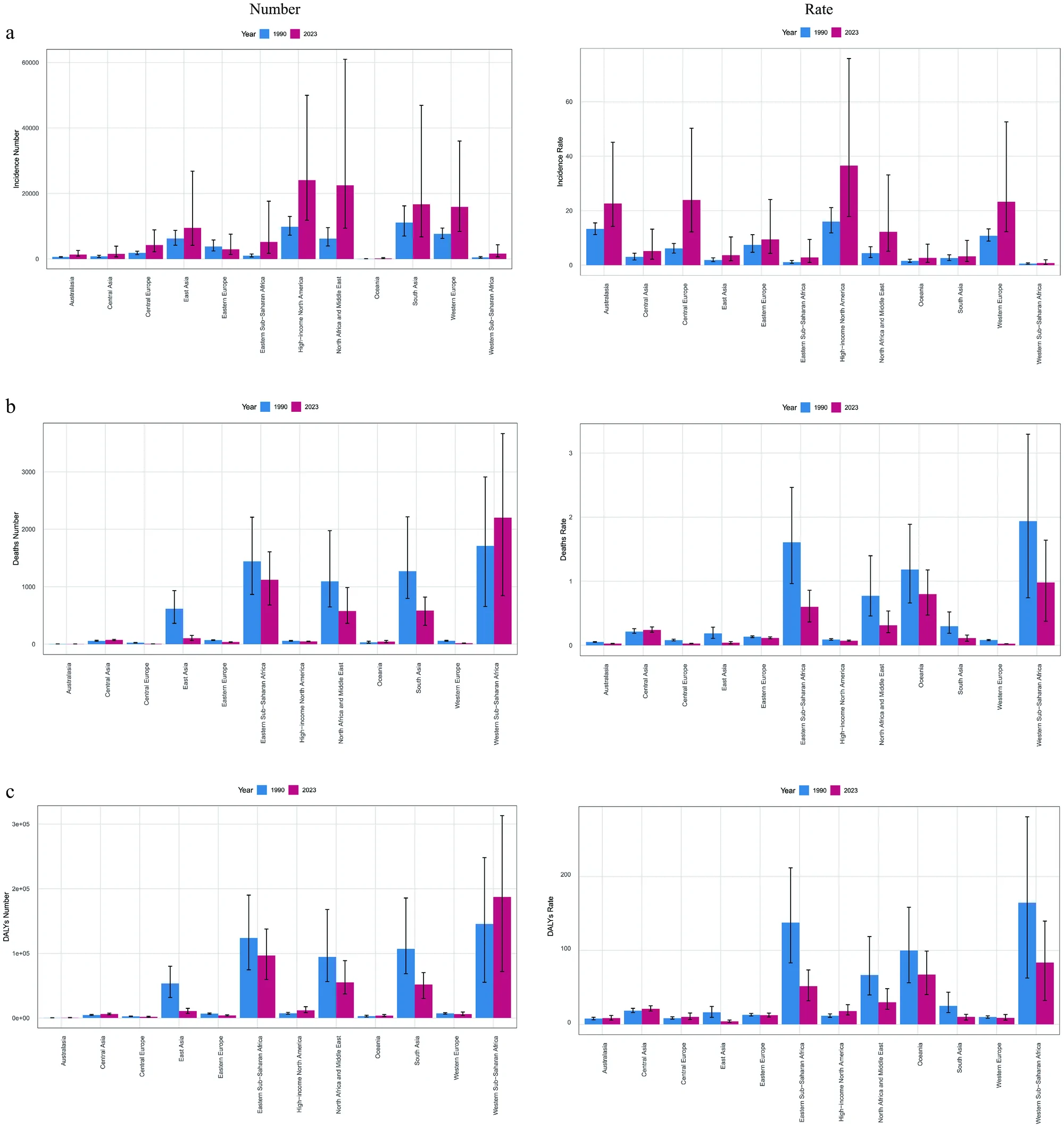
Regional burden of childhood diabetes (ages 0–14 years) in 1990 and 2023. (a) Number of incident cases and incidence rate (per 100,000 population); (b) Number of deaths and mortality rate; (c) Number of disability-adjusted life years (DALYs) and DALY rate (per 100,000 population).

Sub-Saharan Africa remained the hotspot for childhood diabetes mortality and disability burden. Western Sub-Saharan Africa reported the highest number of deaths in 2023, reaching 2,202 (845–3,670), along with the highest mortality rate of 0.98 (0.38–1.64) per 100,000 (Fig. 1; Supplementary Fig. S1 and Supplementary Table S2). Mortality was greatest among children under 5 years, and boys exhibited slightly higher rates than girls across all age groups (Supplementary Figs S2, S3). During 2019–2023, the number of deaths increased most rapidly in Oceania, whereas Central Asia recorded the fastest rise in ASMR (Supplementary Table S2). Spearman correlation analysis demonstrated a significant negative association between SDI and mortality (r = –0.83, *p* < 0.001; *n* = 84), indicating that higher SDI values were associated with lower death rates (Supplementary Fig. S4).

Similarly, Sub-Saharan Africa exhibited the greatest DALYs burden, with Western Sub-Saharan Africa recording 187,188 (71,993–313,001) DALYs and an ASDR of 83.58 (32.15–139.76) per 100,000 in 2023 (Fig. 1; Supplementary Fig. S1 and Supplementary Table S3). The DALYs burden was concentrated among children under 5 years, with boys more affected than girls (Supplementary Figs S2, S3). High-income North America showed the fastest increase in both DALYs and ASDR between 2019 and 2023 (Supplementary Table S3). SDI remained negatively correlated with DALYs (r = –0.81, *p* < 0.001; *n* = 84), highlighting the persistent inequality between socioeconomic development and disease burden (Supplementary Fig. S4).

### Key regional exemplars

Given that high-income North America exhibited the highest incidence, we further examined its temporal patterns. From 1990 to 2023, joinpoint regression identified four distinct phases. The ASIR rose sharply from 1990 to 2000 (APC, 4.98; 95% CI, 4.81–5.17) and continued increasing from 2000 to 2010 (APC, 3.60, 3.50–3.72). A temporary decline followed between 2010 and 2015 (APC, –1.39, –1.67 to –1.22), before rebounding modestly after 2015 (APC, 0.61, 0.29–1.00). Sex-stratified analyses revealed similar trajectories for both sexes. Among females, ASIR rose during 1990–1999 (APC, 5.15), 1999-2004 (APC, 4.08), and 2004–2010 (APC, 3.03), declined from 2010–2015 (APC, –1.38), and then stabilized (APC, 0.20). Males displayed comparable phases, with marked increases from 1990–1999 (APC, 4.85) and 1999–2010 (APC, 3.86), followed by a decline (APC, –1.27) and slight rebound (APC, 0.94). Across the entire period, the AAPC remained significantly positive (2.51, 2.44–2.57; *p* < 0.001), slightly higher among males (2.62) than females (2.36). Despite transient reductions in the early 2010s, the long-term trend indicates a persistent rise in incidence across High-income North America (Supplementary Fig. S5a).

Western Sub-Saharan Africa, which bore the highest mortality and DALYs burden, showed a markedly different pattern. Both ASMR and ASDR exhibited a single joinpoint in the early 2000s, signaling an accelerated decline. The ASMR decreased moderately during 1990–1999 (APC, –1.08, –1.36 to –0.73) and more steeply thereafter (APC, –2.31, –2.38 to –2.24). A similar trend was observed for ASDR (APC, –1.07 early; –2.30 after 1999). Females experienced a slower initial reduction, whereas males showed a steeper and more consistent decline. Overall, both ASMR and ASDR fell significantly (AAPC, –1.97 and –1.96; *p* < 0.001), indicating sustained improvement in survival and disability outcomes despite limited resources (Supplementary Fig. S5b–S5c).

### Key national exemplars

Substantial national heterogeneity was also observed. In earlier GBD datasets (2019 and 2021), India reported the highest number of new childhood diabetes cases, but in 2023, the United States ranked first, with 21,960 (10,641–45,402) new cases. Finland recorded the highest incidence rate at 59.84 (39.13–106.02) per 100,000. From 1990 to 2023, Qatar, Chile, and South Korea showed the fastest increases in incidence (EAPCs 5.85, 5.58, and 5.47), while Poland had the largest rise in Central Europe (EAPC 4.89) (Fig. 2). Nigeria had the highest mortality in 2023, both in number (1,161; 338–2,079) and rate (1.96; 0.83–3.36), whereas Uzbekistan exhibited the steepest increase in Central Asia (EAPC 1.01) (Supplementary Fig. S6). In 2023, Nigeria had the highest DALYs (98,959; 28,787–177,686), and Niger surpassed Tokelau with the highest DALYs rate (166.21; 70.03–283.25). From 1990 to 2023, Poland showed the largest DALYs increase (EAPC 2.25), while the United States had the highest rise among high-income regions (EAPC 1.40) (Supplementary Fig. S7).

**Figure 2:**
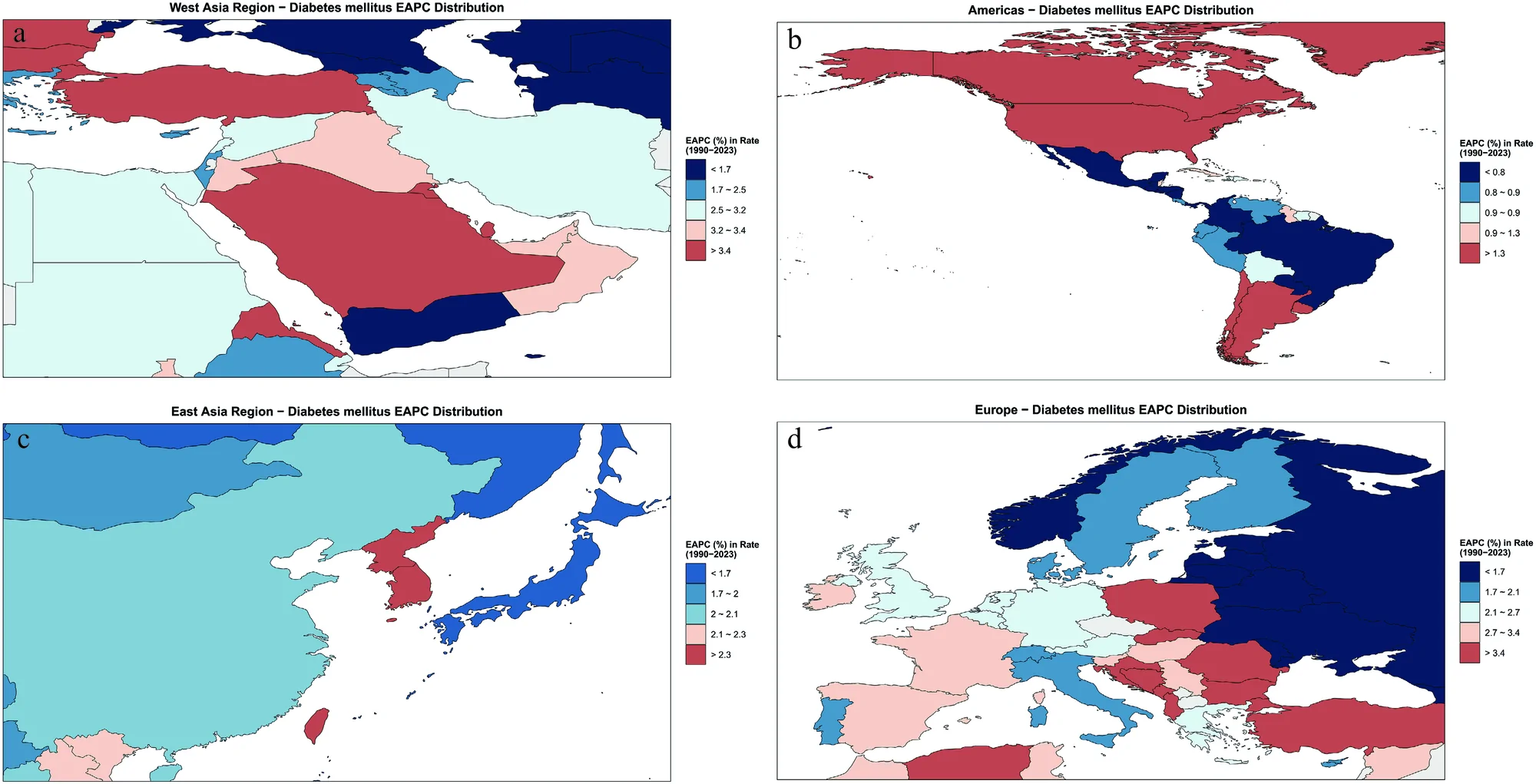
National distribution of the estimated annual percentage change (EAPC) in the incidence rate of childhood diabetes from 1990 to 2023. Map created using a base map from the Resource and Environment Science and Data Center (RESDC, www.resdc.cn) and data from the GBD Study 2023. (a) West Asia; (b) the Americas; (c) East Asia; (d) Europe. EAPC reflects the average annual change in incidence rate over time, with red indicating an increasing trend and blue indicating a declining trend.

### Risk factor analysis

Childhood diabetes mortality was primarily driven by metabolic (high fasting plasma glucose, HFPG) and environmental risks (low and high temperatures). In 2023, HFPG-related mortality was highest in Western Sub-Saharan Africa and increased most in Central Asia. High-temperature mortality was concentrated in Western Sub-Saharan Africa and rose fastest in Eastern Europe, whereas low-temperature mortality peaked in Oceania but declined elsewhere, with minimal reduction in Central Asia. From 1990 to 2023, East Asia achieved the largest decreases across all three risk categories (Fig. 3). These results suggest that metabolic and environmental risk-attributable mortality patterns may contribute to regional differences in childhood diabetes deaths.

**Figure 3:**
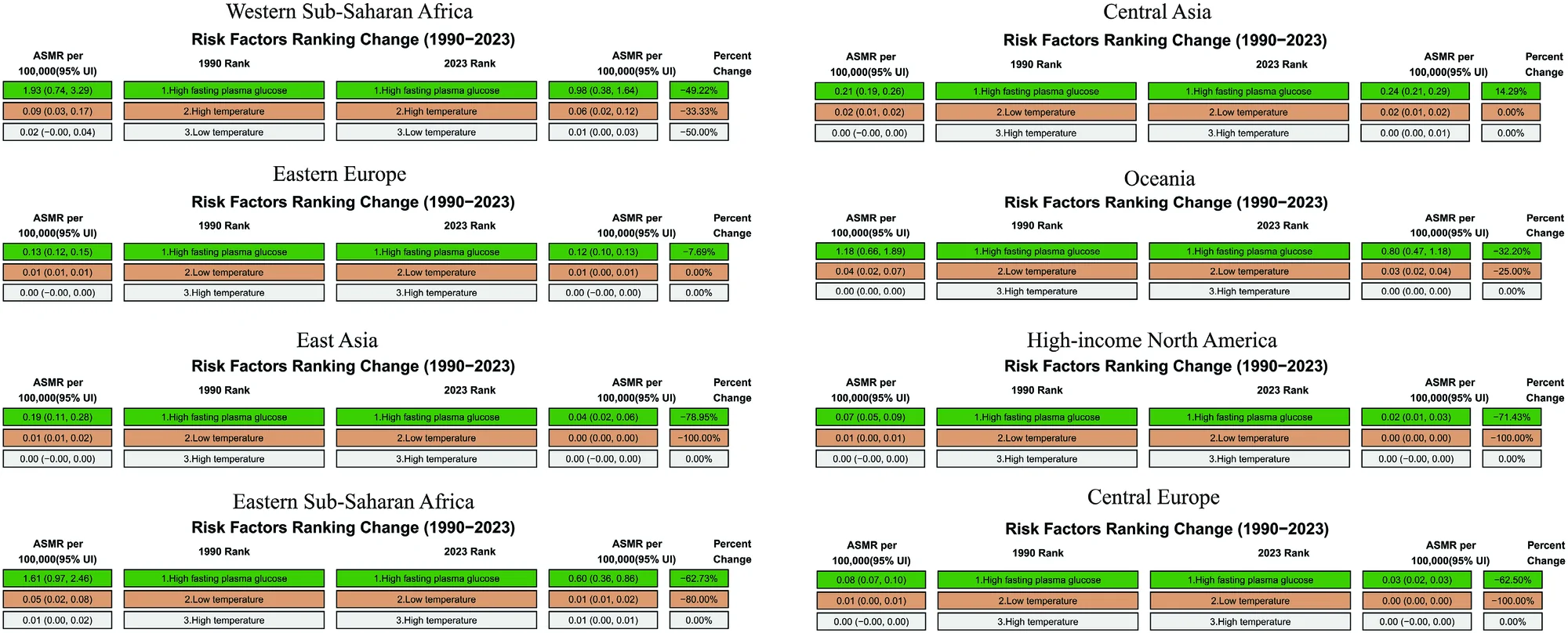
Changes in risk factor–attributable burden of childhood diabetes deaths across selected regions from 1990 to 2023.

### Quality of childhood diabetes care

QCI was used to assess whether higher mortality and disability coincided with weaker care-related performance. In 2023, marked disparities in the QCI for childhood diabetes were observed across regions. High-Income North America showed uniformly high QCI values (> 70), consistent with better care-related performance, including diagnosis, survival, and long-term management. In contrast, Western Sub-Saharan Africa exhibited substantially lower and more heterogeneous QCI scores (20–70), indicating persistent inequities in pediatric diabetes care (Fig. 4).

**Figure 4:**
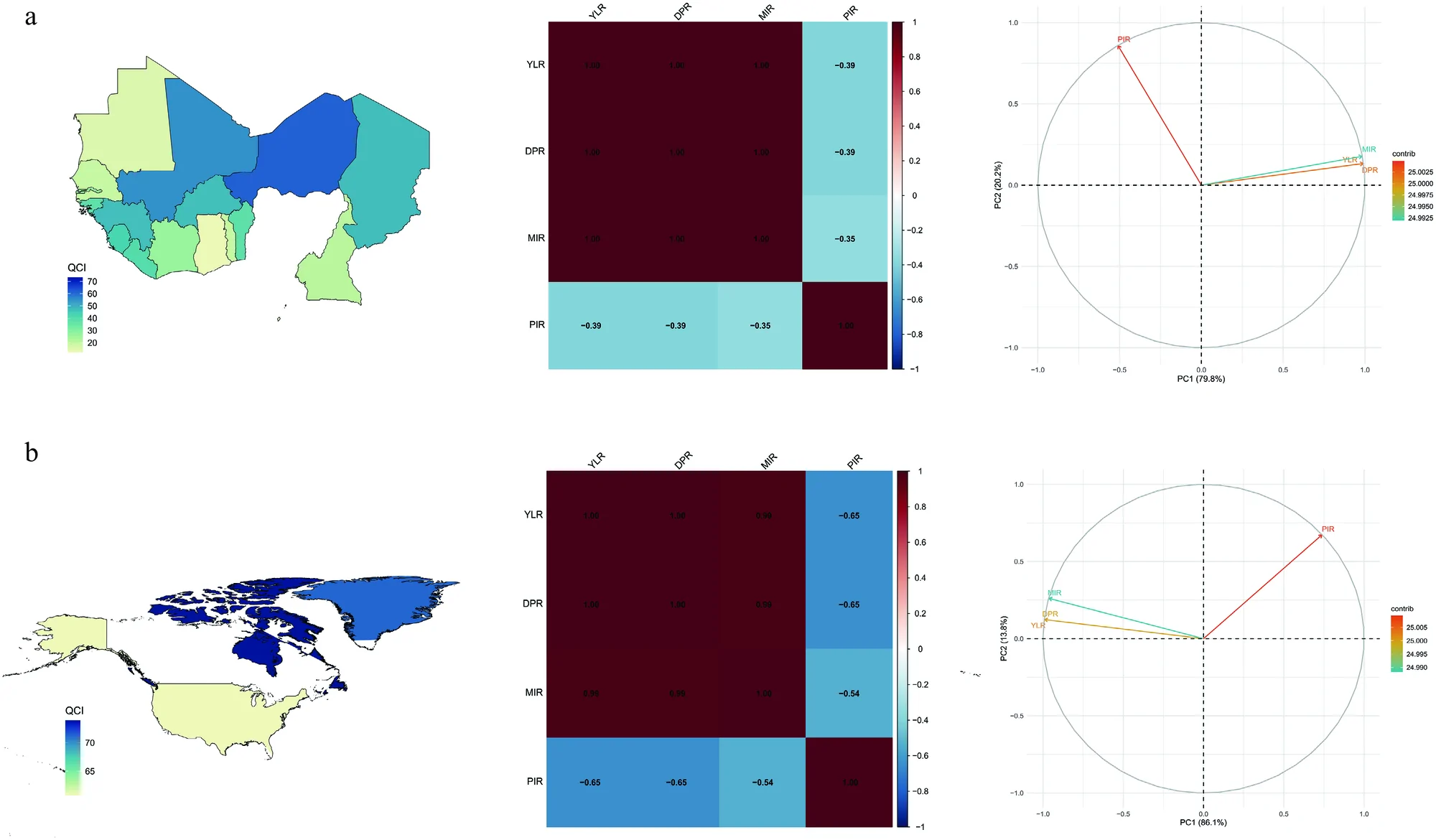
Regional quality of childhood diabetes care based on the Quality of Care Index (QCI) in Western Sub-Saharan Africa (a) and High-Income North America (b). Distribution of QCI; correlation matrix illustrating the relationships among YLR, DPR, MIR, and PIR; and principal component analysis identifying two major components representing disease burden (PC1) and management quality (PC2).

In Western Sub-Saharan Africa, YLR, DPR, and MIR were strongly correlated (r = 0.998–1.000; *p* < 0.001), while PIR showed weaker inverse associations (r = −0.35 to −0.39; *p* = 0.01). PCA explained 99.98% of total variance, with PC1 (79.82%) representing disease burden and PC2 (20.16%) reflecting survival. These findings underscore pronounced disparities between acute and chronic care quality across regions.

In High-Income North America, YLR, DPR, and MIR were again highly correlated (r = 0.989–1.000; *p* < 0.001), forming a common disease burden dimension. PIR was moderately and inversely correlated (r = −0.54 to −0.65; *p* < 0.001), representing survival and chronic care. PCA identified two components explaining 99.96% of variance: PC1 (86.13%), defined by YLR, DPR, and MIR, indicating overall care performance, and PC2 (13.83%), defined by PIR, reflecting long-term management quality.

### EAPC–HDI association

Analysis of EAPC in relation to the HDI revealed no significant linear correlations for ASIR, ASMR, or ASDR (all *p* > 0.05). EAPC in ASIR showed a weak negative correlation (r = –0.25, *p* = 0.16), suggesting slower incidence growth in higher-HDI countries. ASMR exhibited minimal association (r = –0.07, *p* = 0.71), while ASDR displayed a slight U-shaped pattern (r = 0.14, *p* = 0.43), with sharper declines in moderate-HDI regions and mild rebounds in high-HDI areas. These findings underscore heterogeneous, non-linear relationships between socioeconomic development and the evolving childhood diabetes burden (Supplementary Fig. S8).

### Supportive SHAP-based summary of demographic and temporal contributions

SHAP analysis was used as a supportive tool to summarize the relative contribution of basic demographic and temporal variables, rather than to identify causal determinants. The XGBoost SHAP model showed distinct regional patterns in predictor contribution. In Western Sub-Saharan Africa, age contributed most strongly to predicted incidence and DALY rates (Fig. 5a). Older children aged 10–14 years had higher predicted incidence and disability burdens, and calendar year showed a gradual temporal contribution. These patterns may be consistent with delayed diagnosis, improved ascertainment, or changing access to care, but they should not be interpreted as direct causal evidence. In High-Income North America, age and year contributed most strongly to predicted incidence and DALY rates, whereas sex and population size contributed less (Fig. 5b). Overall, age and temporal variables were consistent contributors to model predictions across regions.

**Figure 5:**
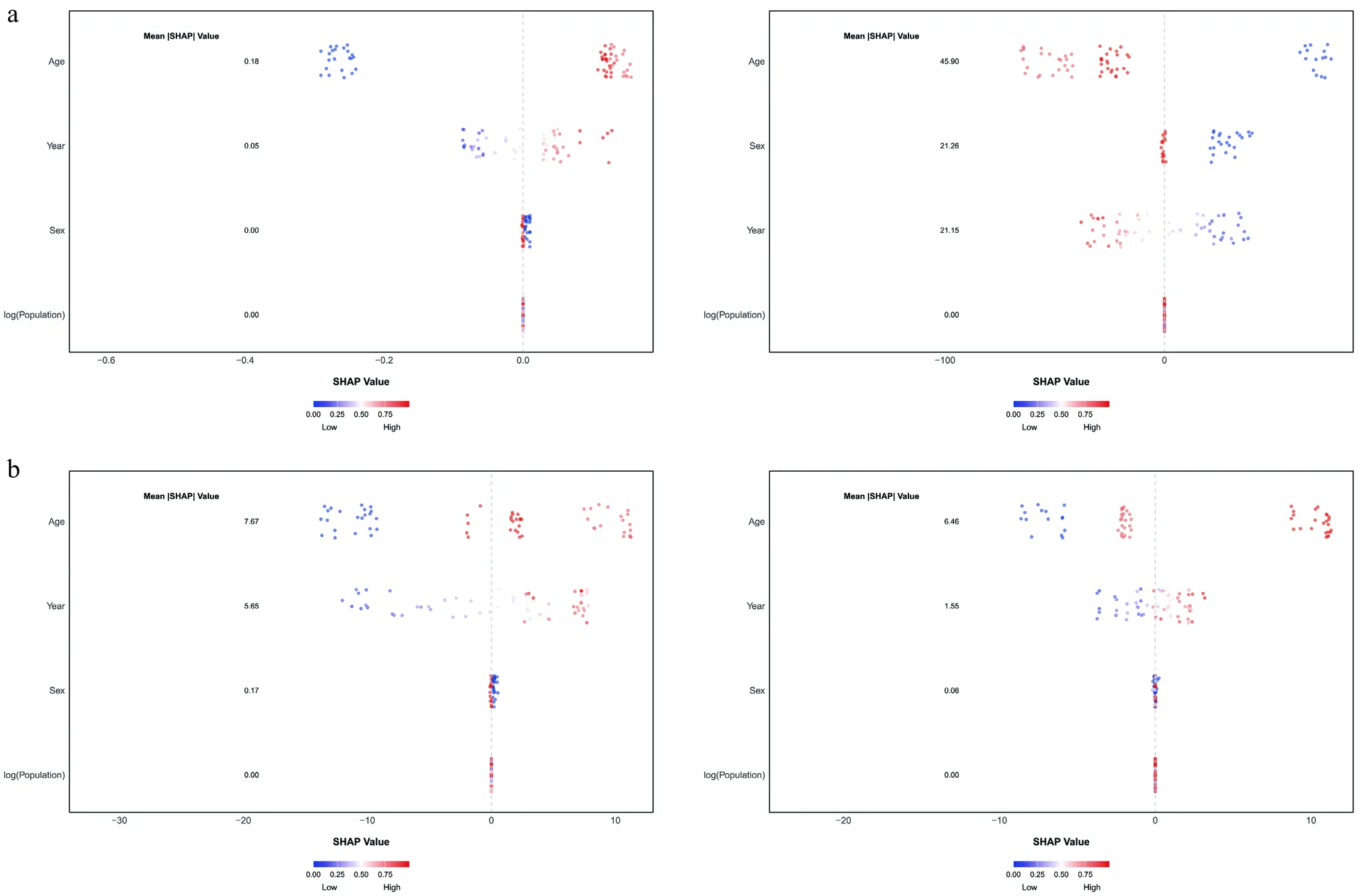
SHAP-based interpretation of childhood diabetes incidence and DALY Rates in Western Sub-Saharan Africa and High-Income North America. (a) Western Sub-Saharan Africa. (b) High-income North America.

### Forecast of childhood diabetes incidence

Forecast analyses of high- and representative-incidence countries revealed divergent trends. The United States is projected to peak in 2030 with an ASIR of 37.97 per 100,000, followed by a gradual decline to 35.89 by 2045. In contrast, China, India, Poland, Finland, and Qatar are expected to continue rising, reaching 5.31, 4.42, 42.50, 55.55, and 106.56 per 100,000, respectively, indicating widening epidemiologic disparities (Supplementary Fig. S9).

## Discussion

This GBD 2023-based analysis focused on regional inequalities in childhood diabetes burden from 1990 to 2023 and their potential persistence into the future. We identified a persistent dual inequality pattern: childhood diabetes incidence increased in most regions, whereas mortality and DALYs declined overall. However, these changes were uneven across regions. High-Income North America had the highest incidence in 2023, consistent with broader registry coverage and improved case ascertainment, whereas Western Sub-Saharan Africa continued to bear the greatest mortality and disability burden. These findings suggest that high-detection settings face an expanding diagnosed burden, while resource-limited settings continue to experience higher mortality and disability.

These results extend earlier GBD estimates by clarifying shifting geographic and socioeconomic patterns in childhood diabetes burden. South Asia’s relative contribution to incidence appeared lower in the GBD 2023 estimates, whereas Central and Eastern Europe, the Middle East, and several Asian economies showed increasing incidence trends. Finland continued to report the highest incidence, while Qatar, Chile, and South Korea showed rapid increases. These patterns may be related to urbanization, lifestyle transition, environmental exposure, improved diagnostic capacity, and differences in registry coverage^[23−25]^. Persistently high mortality in Nigeria and Niger may reflect weaker health-system capacity, limited access to pediatric diabetes care, and socioeconomic constraints. Socioeconomic development showed a dual association with disease burden: SDI was positively correlated with incidence but negatively correlated with mortality and DALYs. This pattern suggests that higher-SDI regions may achieve broader diagnosis and better survival, whereas lower-SDI regions may experience greater diagnostic and treatment gaps. Emerging and transitional economies such as China, India, and Poland showed increasing incidence trends, which may reflect a combination of improved ascertainment, demographic change, lifestyle transition, and evolving metabolic risk profiles.

The rise in incidence may reflect the convergence of environmental, lifestyle, and health-system factors. The 2021–2023 period should be interpreted cautiously because post-pandemic changes may reflect several overlapping processes. Part of the observed increase may reflect catch-up diagnosis after delays in presentation, disrupted routine care, and reduced healthcare utilization during the early pandemic period. COVID-19-related viral infection, lifestyle disruption, and psychosocial stress have also been proposed as potential contributors to childhood diabetes onset^[26]^. At the same time, recovery of health services, heightened health awareness, and expanded use of telemedicine may have improved case ascertainment after 2021^[5,11,27−32]^. The steady decline in mortality and DALYs may be consistent with progress in diabetes care, including wider insulin availability, improved management of diabetic ketoacidosis, and expanded glucose monitoring^[33−35]^. However, persistently high mortality in Sub-Saharan Africa and Central Asia suggests that these gains remain uneven. Limited insulin access, inadequate clinician training, and weak healthcare infrastructure may continue to constrain progress. Sustainable improvement will require resilient pediatric diabetes systems that ensure accessibility, affordability, and continuity of care.

The QCI analysis provides additional context by translating mortality, disability, and incidence-related indicators into a care-related inequality framework. Rather than serving as a separate conclusion, QCI supports the main finding by assessing whether regions with higher mortality and disability also show weaker care-related performance. High-Income North America showed higher QCI values, consistent with stronger diagnosis, survival, and long-term management, whereas Western Sub-Saharan Africa showed lower and more variable scores. These findings suggest that survival gains may depend not only on disease occurrence but also on early recognition, reliable insulin access, acute complication management, and continuity of pediatric diabetes care^[36−38]^.

The SHAP analysis was included to support the interpretation of broad demographic and temporal patterns. Its added value lies in summarizing how basic demographic and temporal variables contributed to model predictions beyond descriptive trend analyses. Age and calendar year contributed more strongly than sex or population size in the selected regional models, suggesting that age structure and temporal changes were closely aligned with predicted incidence and DALY patterns. However, because the model included only year, age, sex, and population size, SHAP findings should be interpreted as summaries of predictor contribution rather than evidence of biological mechanisms or causal determinants.

Forecast analyses suggest divergent future trajectories. The United States may reach a plateau by 2030, possibly reflecting stabilization of recent incidence trends and relatively mature diagnostic systems. In contrast, China, India, Poland, Finland, and Qatar are projected to continue rising through 2045, suggesting that future incidence inequalities may remain geographically heterogeneous. These projections should be interpreted as extensions of historical trends rather than definitive predictions, because future changes in surveillance, prevention, diagnosis, and treatment access may alter these trajectories. Priorities include early diagnosis, reliable insulin access, diabetes education, and strengthened pediatric diabetes services.

Despite the strengths of the GBD framework, this study remains constrained by the inherent limitations of modeled estimates. Variations in case definitions, registry completeness, coding practices, and data quality may affect cross-country comparisons, especially in low-income and data-sparse settings where estimates rely more heavily on statistical modeling and often have wider 95% UIs. Therefore, precise differences in magnitude and regional rank order should be interpreted cautiously, and underdiagnosis in resource-limited settings may lead to underestimation of incidence. In addition, risk attribution, QCI, SHAP, and forecasting analyses were supportive, population-level analyses and should not be interpreted as evidence of individual-level causality or biological mechanisms. BAPC projections were based on historical trends and may not capture future changes in prevention, diagnosis, treatment access, or health policy. Future work incorporating high-quality registry data, longitudinal cohorts, and health-system indicators will be important to refine estimates and better characterize regional inequalities.

## Conclusions

Childhood diabetes incidence continues to rise despite overall declines in mortality and disability. The GBD 2023 analysis revealed persistent regional and socioeconomic disparities, with High-Income North America showing the highest incidence and Western Sub-Saharan Africa bearing the greatest mortality and disability burden. Supportive analyses suggested that these inequalities were accompanied by care-related performance gaps and divergent future incidence trajectories. Strengthening early detection, reliable insulin access, acute complication management, and continuity of pediatric diabetes care is essential to reduce preventable mortality and disability in resource-limited settings.

## SUPPLEMENTARY DATA

Supplementary data to this article can be found online.

## Data Availability

The data that support the findings of this study are available in the Global Burden of Disease Study 2023 (GBD 2023) repository. These data were derived from publicly available resources provided by the Global Health Data Exchange: https://ghdx.healthdata.org/gbd-2023.
