# Supplementary material for: Diverging regional inequalities in childhood diabetes, 1990–2023: a GBD 2023-based analysis
Source: Pediatr Diabetes. 2026 Jun 11;2026:e001. doi: 10.48130/pedi-0026-0002 (PMC13463378; doi:10.48130/pedi-0026-0002)
Supplement: Supplementary file 1 [file pedi-2026-2026-1-0002-S1.zip › 10.48130_pedi-0026-0002-Suppl-FigureS1.pdf]

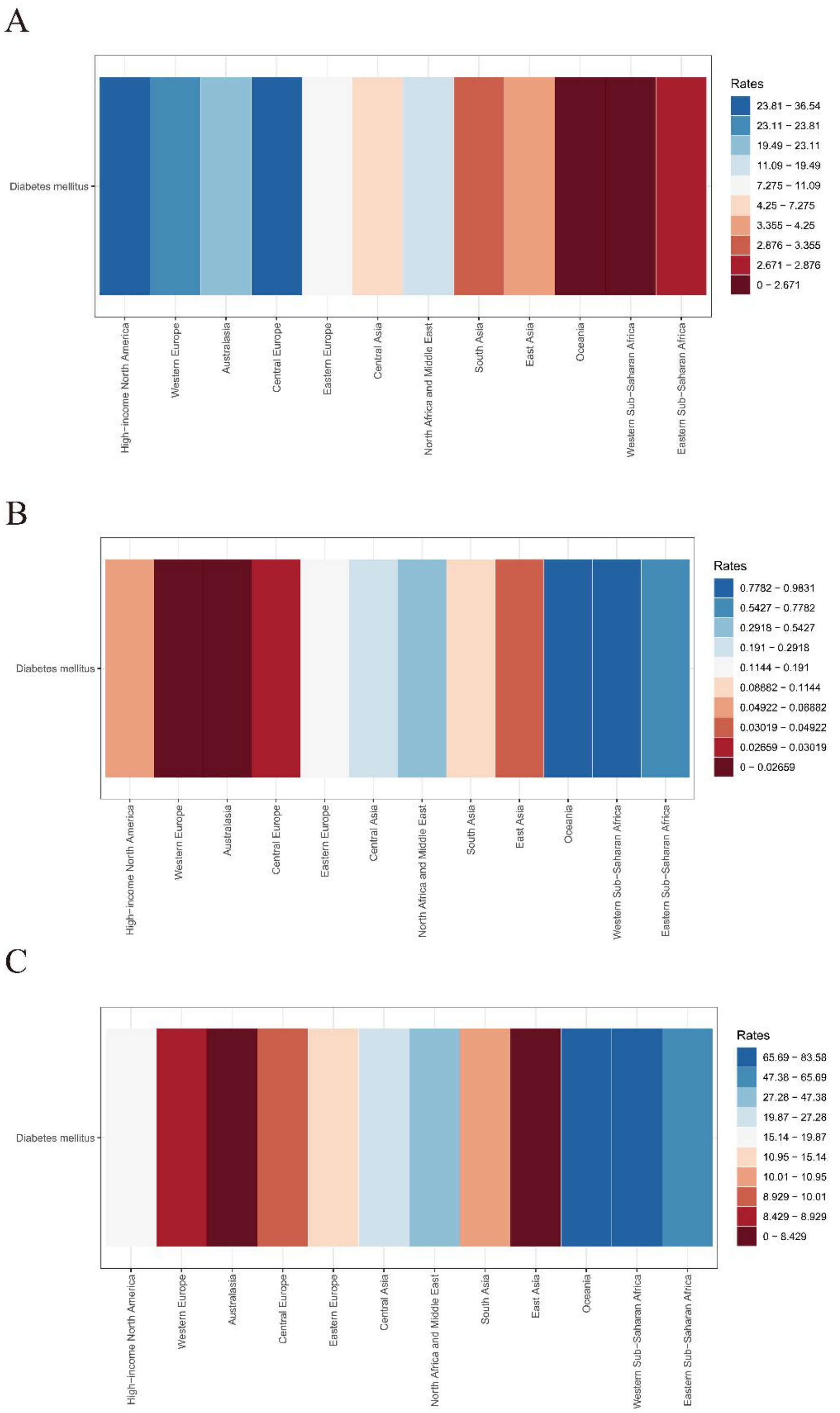

Appendix Figure 1. Regional heatmap of childhood diabetes burden in 2023. (A) Incidence rate (per 100,000 population); (B) Mortality rate; (C) DALY rate. Colors represent relative magnitude, with darker red indicating higher rates and darker blue indicating lower rates.
