# Supplementary material for: Diverging regional inequalities in childhood diabetes, 1990–2023: a GBD 2023-based analysis
Source: Pediatr Diabetes. 2026 Jun 11;2026:e001. doi: 10.48130/pedi-0026-0002 (PMC13463378; doi:10.48130/pedi-0026-0002)
Supplement: Supplementary file 1 [file pedi-2026-2026-1-0002-S1.zip › 10.48130_pedi-0026-0002-Suppl-FigureS2.pdf]

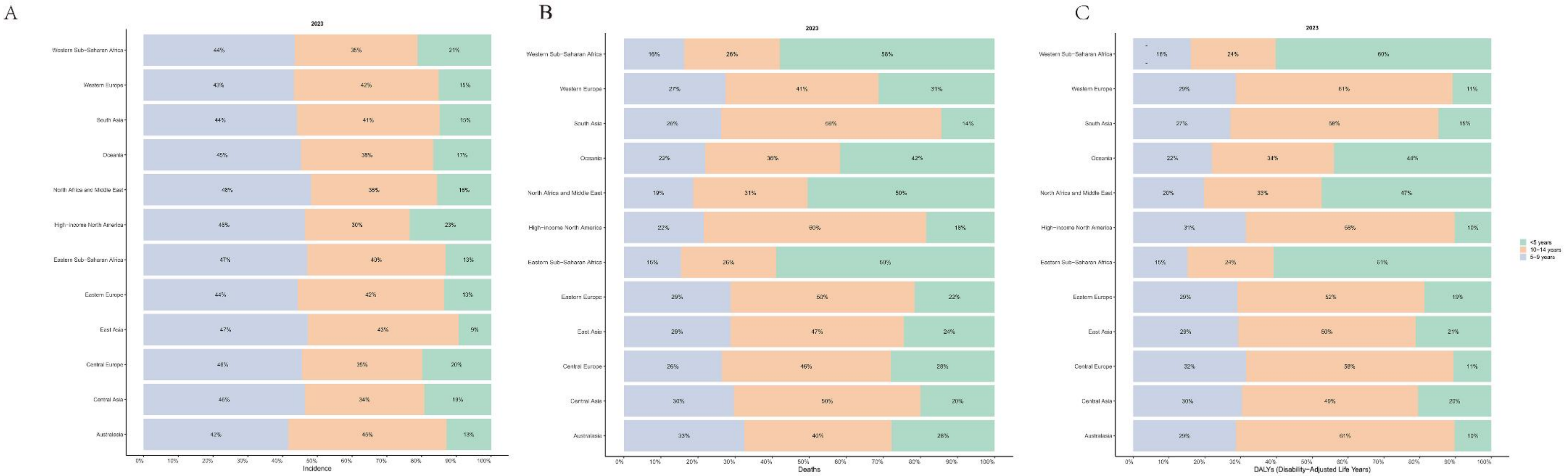

Appendix Figure 2. Age-specific proportional distribution of childhood diabetes burden across GBD regions in 2023. (A) Proportional distribution of incident childhood diabetes cases across age groups (<5, 5–9, and 10–14 years) in 21 GBD regions; (B) Proportional distribution of deaths across age groups; (C) Proportional distribution of DALY numbers.
