# Supplementary material for: Diverging regional inequalities in childhood diabetes, 1990–2023: a GBD 2023-based analysis
Source: Pediatr Diabetes. 2026 Jun 11;2026:e001. doi: 10.48130/pedi-0026-0002 (PMC13463378; doi:10.48130/pedi-0026-0002)
Supplement: Supplementary file 1 [file pedi-2026-2026-1-0002-S1.zip › 10.48130_pedi-0026-0002-Suppl-FigureS3.pdf]

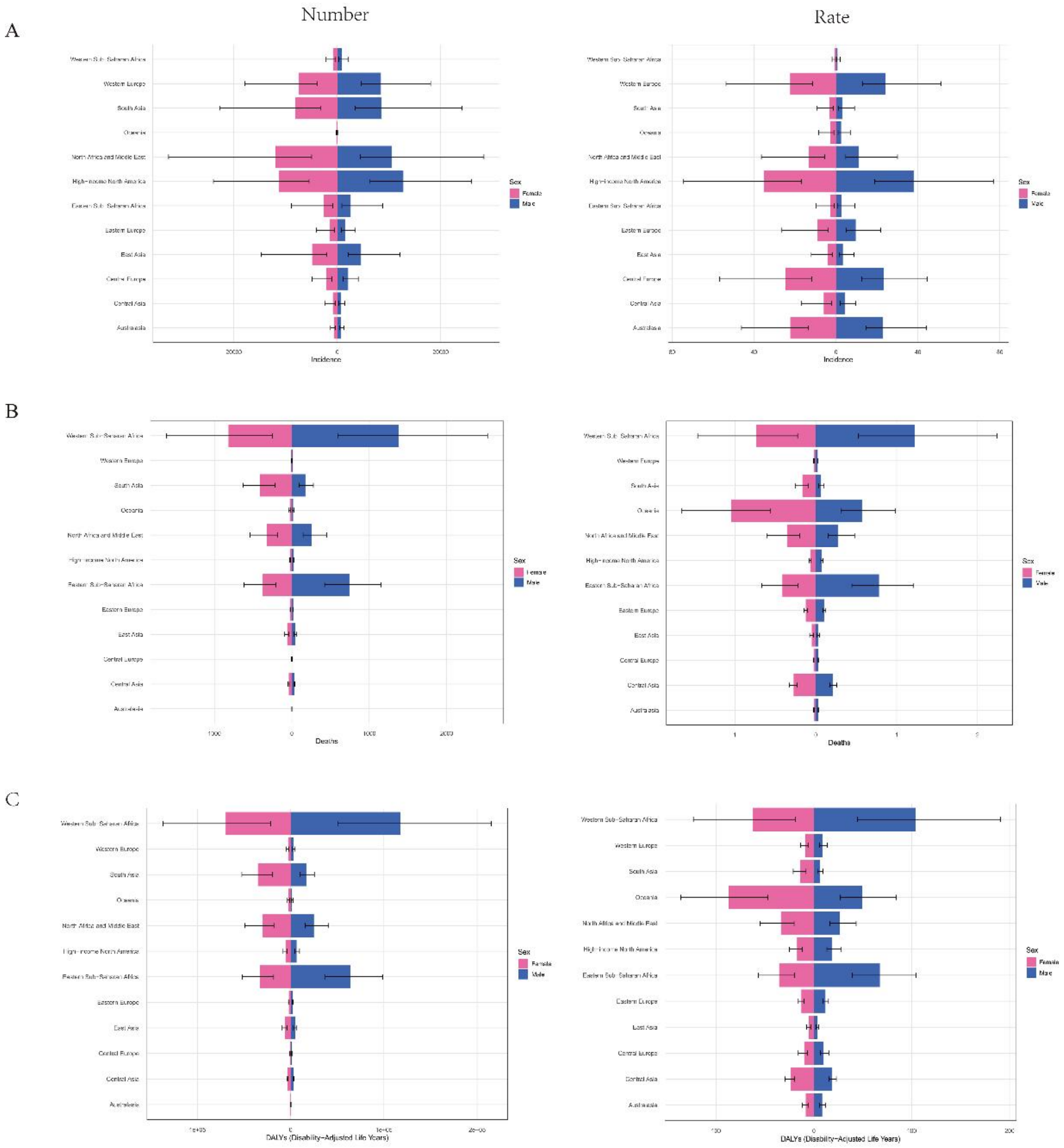

Appendix Figure 3. Sex-specific burden of childhood diabetes across GBD regions in 2023. (A) Number and rate of incident cases by sex; (B) Number and rate of deaths by sex; (C) Number and rate of DALYs by sex. Pink and blue bars represent females and males, respectively.
