# Supplementary material for: Diverging regional inequalities in childhood diabetes, 1990–2023: a GBD 2023-based analysis
Source: Pediatr Diabetes. 2026 Jun 11;2026:e001. doi: 10.48130/pedi-0026-0002 (PMC13463378; doi:10.48130/pedi-0026-0002)
Supplement: Supplementary file 1 [file pedi-2026-2026-1-0002-S1.zip › 10.48130_pedi-0026-0002-Suppl-FigureS4.pdf]

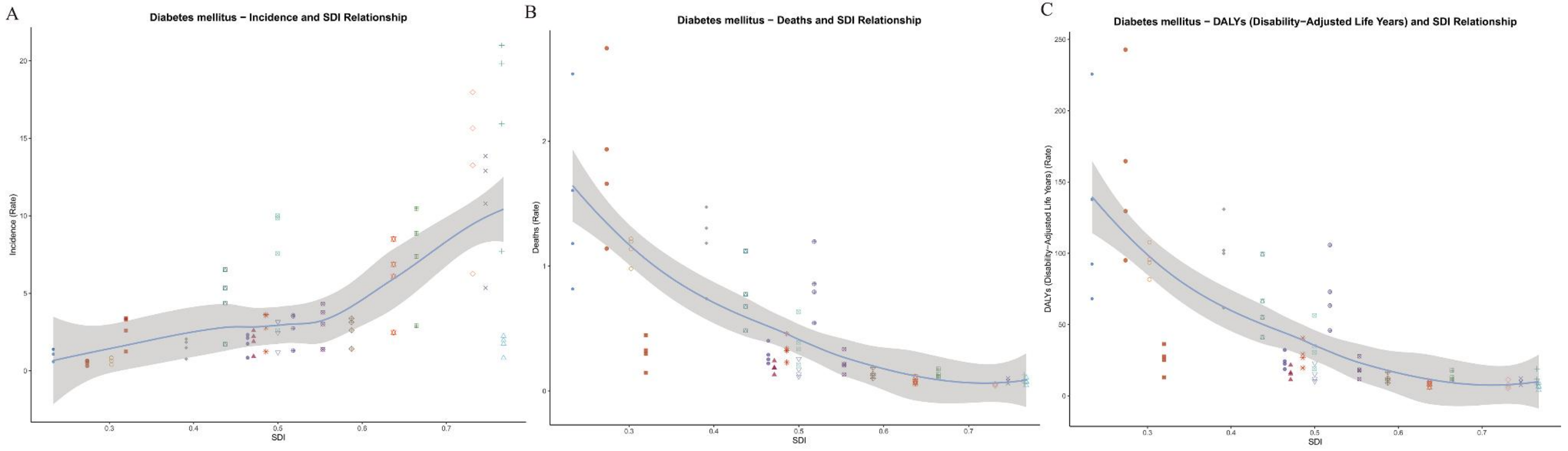

Appendix Figure 4. Association between Socio-demographic Index (SDI) and childhood diabetes burden in 2023. (A) Relationship between SDI and incidence rate; (B) Relationship between SDI and mortality rate; (C) Relationship between SDI and DALYs rate.
