# Supplementary material for: Diverging regional inequalities in childhood diabetes, 1990–2023: a GBD 2023-based analysis
Source: Pediatr Diabetes. 2026 Jun 11;2026:e001. doi: 10.48130/pedi-0026-0002 (PMC13463378; doi:10.48130/pedi-0026-0002)
Supplement: Supplementary file 1 [file pedi-2026-2026-1-0002-S1.zip › 10.48130_pedi-0026-0002-Suppl-FigureS5.pdf]

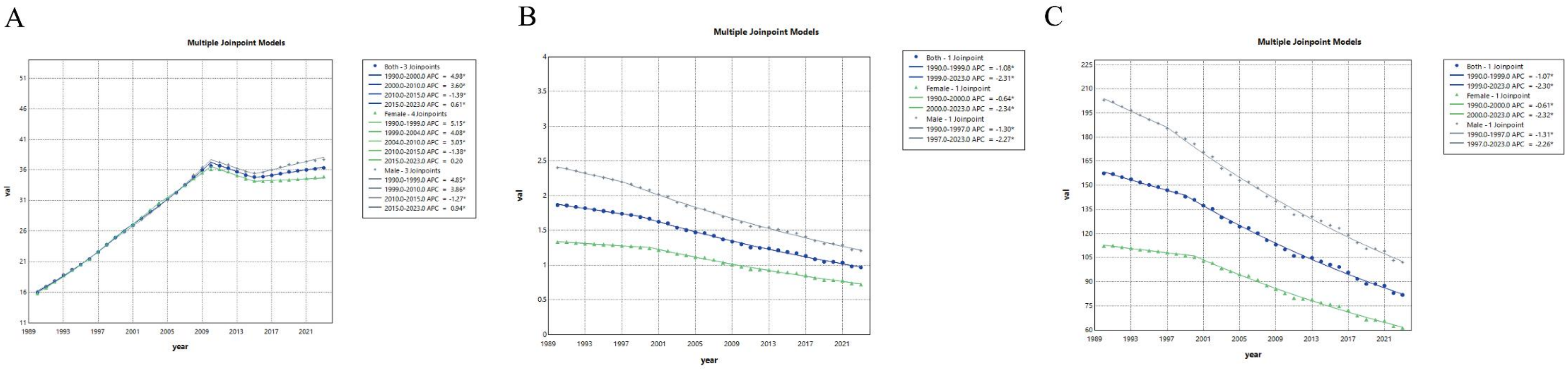

Appendix Figure 5. Temporal trends of childhood diabetes in representative regions from 1990 to 2023 based on joinpoint regression analysis. (A) Temporal trends and annual percent change (APC) in age-standardized incidence rate (ASIR) in High-income North America; (B) Temporal trends and APC in age-standardized mortality rate (ASMR) in Western Sub-Saharan Africa; (C) Temporal trends and APC in age-standardized DALY rate (ASDR) in Western Sub-Saharan Africa.
