# Supplementary material for: Diverging regional inequalities in childhood diabetes, 1990–2023: a GBD 2023-based analysis
Source: Pediatr Diabetes. 2026 Jun 11;2026:e001. doi: 10.48130/pedi-0026-0002 (PMC13463378; doi:10.48130/pedi-0026-0002)
Supplement: Supplementary file 1 [file pedi-2026-2026-1-0002-S1.zip › 10.48130_pedi-0026-0002-Suppl-FigureS8.pdf]

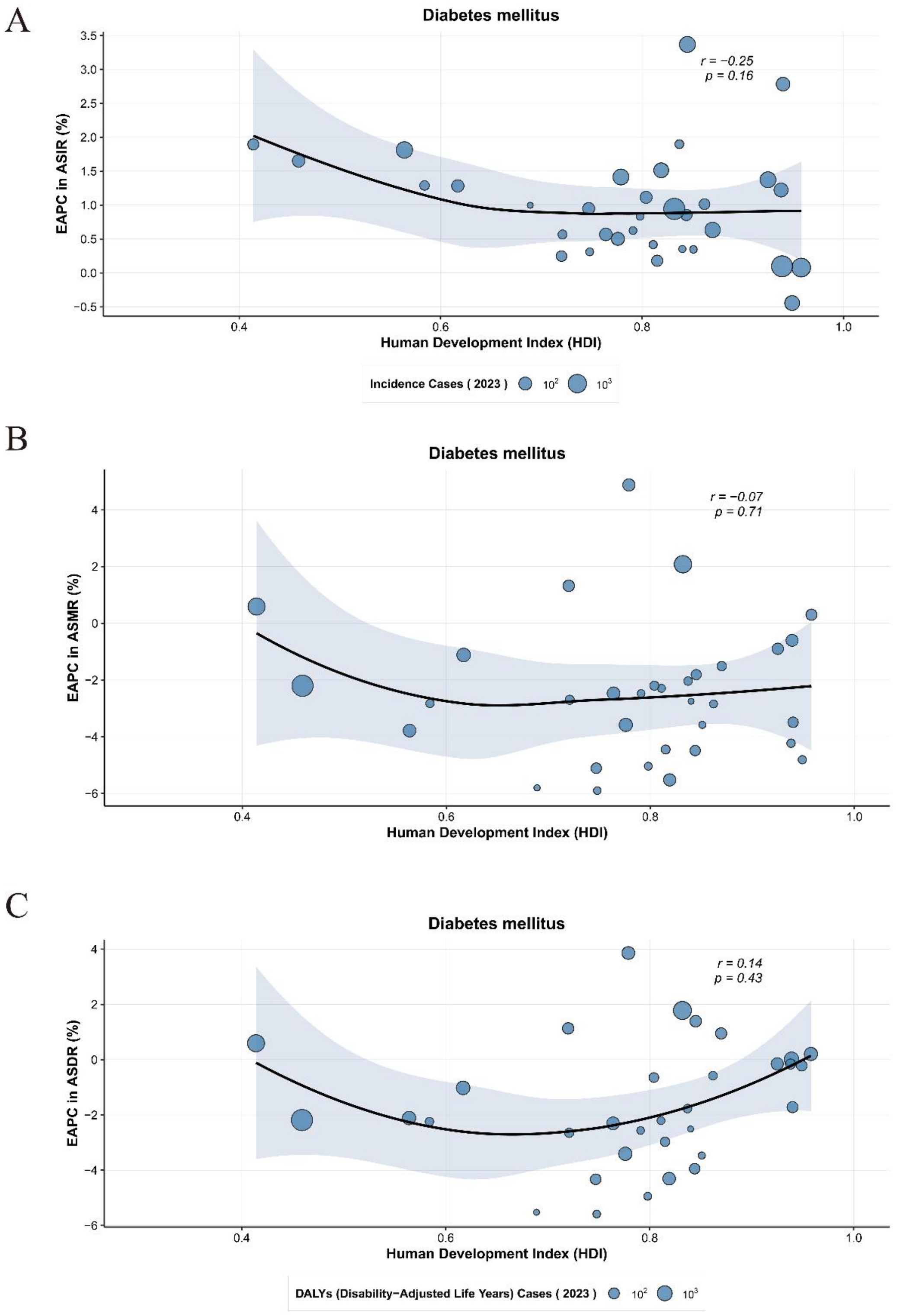

Appendix Figure 8. Correlation between Human Development Index (HDI) and temporal trends in childhood diabetes burden from 1990 to 2023.(A) Association between HDI and EAPC in incidence rate; (B) Association between HDI and EAPC in mortality rate; (C) Association between HDI and EAPC in DALY rate.
