# Supplementary material for: Diverging regional inequalities in childhood diabetes, 1990–2023: a GBD 2023-based analysis
Source: Pediatr Diabetes. 2026 Jun 11;2026:e001. doi: 10.48130/pedi-0026-0002 (PMC13463378; doi:10.48130/pedi-0026-0002)
Supplement: Supplementary file 1 [file pedi-2026-2026-1-0002-S1.zip › 10.48130_pedi-0026-0002-Suppl-Method1.pdf]

Appendix Method 1:Regional and National Classification

GBD Super Regions and Their Constituent Region

GBD Super Region	Constituent Regions	Representative Countries
**High-income**	High-income Asia Pacific, High-income North America, Western Europe, Southern Latin America, Australasia	United States, Canada, Japan, South Korea, Germany, Australia
**Central Europe, Eastern Europe, and Central Asia**	Central Europe, Eastern Europe, Central Asia	Poland, Czech Republic, Russia, Uzbekistan
**Latin America and Caribbean**	Andean Latin America, Central Latin America, Southern Latin America, Tropical Latin America, Caribbean	Chile, Brazil, Mexico, Cuba
**North Africa and Middle East**	North Africa, Middle East	Qatar, Egypt, Iran, Saudi Arabia
**South Asia**	South Asia	India, Bangladesh, Nepal, Pakistan
**Southeast Asia, East Asia, and Oceania**	Southeast Asia, East Asia, Oceania	China, Japan, Vietnam, Indonesia, Australia
**Sub-Saharan Africa**	Western Sub-Saharan Africa, Eastern Sub-Saharan Africa, Central Sub-Saharan Africa, Southern Sub-Saharan Africa	Nigeria, Niger, Kenya, South Africa

GBD Super-Regional and Regional Classification of Selected Countries

Country	GBD Super Region	Region
**Qatar**	North Africa and Middle East	Middle East
**Chile**	Latin America and Caribbean	Southern Latin America
**South Korea**	High-income	High-income Asia Pacific
**Poland**	Central Europe, Eastern Europe, and Central Asia	Central Europe
**United States of America**	High-income	High-income North America
**Nigeria**	Sub-Saharan Africa	Western Sub-Saharan Africa
**Niger**	Sub-Saharan Africa	Western Sub-Saharan Africa
**Uzbekistan**	Central Europe, Eastern Europe, and Central Asia	Central Asia
