# Supplementary material for: Diverging regional inequalities in childhood diabetes, 1990–2023: a GBD 2023-based analysis
Source: Pediatr Diabetes. 2026 Jun 11;2026:e001. doi: 10.48130/pedi-0026-0002 (PMC13463378; doi:10.48130/pedi-0026-0002)
Supplement: Supplementary file 1 [file pedi-2026-2026-1-0002-S1.zip › 10.48130_pedi-0026-0002-Suppl-Method2.pdf]

Appendix Method 2 ： Calculation of the Quality of Care Index

To comprehensively evaluate healthcare quality across countries, we constructed a composite Quality of Care Index (QCI) based on four secondary indicators derived from Global Burden of Disease (GBD) estimates. These metrics indirectly reflect healthcare performance by integrating mortality, disability, and disease persistence dimensions.

The four indicators included:
(1) Years of life lost to years lived with disability ratio (YLR), representing the balance between premature death and life lived with disability. A higher YLR indicates a predominance of early mortality, whereas a lower value implies survival with chronic morbidity;
(2) Disability-adjusted life years to prevalence ratio (DPR), which measures the overall health loss relative to disease prevalence. A higher DPR reflects more severe health consequences despite fewer cases;
(3) Mortality-to-incidence ratio (MIR), serving as a proxy for disease lethality and treatment effectiveness—lower values indicate improved survival and medical management; and
(4) Prevalence-to-incidence ratio (PIR), representing disease chronicity, where higher values suggest longer disease duration or recurrence.

We applied principal component analysis (PCA) to integrate these four indicators into a unified index. PCA extracts orthogonal components that explain the shared variance among indicators. The first principal component (PC1) was used to capture the dominant dimension of variation, summarizing overall quality-of-care performance. To improve robustness, the second component (PC2) was also incorporated, as it accounts for additional variability not captured by PC1.

The four indicators were first calculated as:

YLR = YLLs / YLDs
DPR = DALYs / Prevalence
MIR = Mortality / Incidence
PIR = Prevalence / Incidence

Principal components were derived as:

$PC1 = \omega_1 \times YLR + \omega_2 \times DPR + \omega_3 \times MIR + \omega_4 \times PIR$
$PC2 = \omega_5 \times YLR + \omega_6 \times DPR + \omega_7 \times MIR + \omega_8 \times PIR$

where ω_i represent PCA-derived weights for each variable.

The composite PCA score was then obtained as a weighted combination of the first two components, with weights proportional to the variance each explained:

$$PCA\_score(x) = [Var(PC1) / (Var(PC1) + Var(PC2))] \times PC1 + [Var(PC2) / (Var(PC1) + Var(PC2))] \times PC2$$

Finally, QCI was normalized on a scale of 0 to 100 to facilitate cross-regional comparison:

$$QCI(x) = [(PCA\_score(x) - min(PCA\_score)) / (max(PCA\_score) - min(PCA\_score))] \times 100$$

Higher QCI values indicate more effective healthcare systems with superior diagnosis, treatment, and disease management outcomes.
