# Supplementary material for: Diverging regional inequalities in childhood diabetes, 1990–2023: a GBD 2023-based analysis
Source: Pediatr Diabetes. 2026 Jun 11;2026:e001. doi: 10.48130/pedi-0026-0002 (PMC13463378; doi:10.48130/pedi-0026-0002)
Supplement: Supplementary file 1 [file pedi-2026-2026-1-0002-S1.zip › 10.48130_pedi-0026-0002-Suppl-TableS1.pdf]

Appendix Table 1. Epidemiological Patterns of Childhood Diabetes Incidence Across GBD Regions, 1990-2023

Location	Incidence 1990 (Number)	Incidence 2023 (Number)	Number % Change	Incidence 1990 (Rate)	Incidence 2023 (Rate)	EAPC (95% CI)
Australasia	609.40 (513.90-712.30)	1313.50 (823.60-2619.10)	1.16 (0.16, 4.10)	13.30 (11.20-15.50)	22.60 (14.20-45.10)	1.2 (0.9,1.5)
Central Asia	763.40 (477.70-1114.40)	1525.60 (644.70-3906.40)	1.00 (-0.42, 7.18)	3.00 (1.90-4.40)	5.20 (2.20-13.20)	1.6 (1.4,1.8)
Central Europe	1821.30 (1328.90-2374.00)	4229.20 (2157.60-8900.80)	1.32 (-0.09, 5.70)	6.10 (4.40-7.90)	23.90 (12.20-50.20)	4.7 (4.3,5.1)
Central Sub-Saharan Africa	167.50 (103.50-269.40)	732.60 (250.50-2205.90)	3.37 (-0.07, 20.31)	0.70 (0.40-1.10)	1.20 (0.40-3.50)	1.8 (1.7,1.8)
East Asia	6238.10 (4182.30-8733.50)	9465.10 (4116.70-26816.90)	0.52 (-0.53, 5.41)	1.90 (1.30-2.70)	3.60 (1.60-10.30)	2.1 (2.0,2.3)
Eastern Europe	3822.50 (2443.60-5783.40)	2959.20 (1353.20-7578.10)	-0.23 (-0.77, 2.10)	7.40 (4.70-11.20)	9.40 (4.30-24.10)	0.3 (0.1,0.4)
Eastern Sub-Saharan Africa	963.20 (592.70-1525.10)	5210.00 (1740.60-17632.40)	4.41 (0.14, 28.75)	1.10 (0.70-1.70)	2.80 (0.90-9.40)	3.2 (3.0,3.3)
High-income North America	9842.10 (7291.00-13008.30)	24097.60 (11817.30-49989.40)	1.45 (-0.09, 5.86)	15.90 (11.80-21.10)	36.50 (17.90-75.80)	2.5 (2.1,2.9)
North Africa and Middle East	6185.00 (3967.30-9576.40)	22505.60 (9445.50-60995.90)	2.64 (-0.01, 14.37)	4.40 (2.80-6.80)	12.20 (5.10-33.10)	3.2 (3.0,3.5)
Oceania	40.50 (26.70-58.70)	144.30 (56.30-419.40)	2.56 (-0.04, 14.71)	1.50 (1.00-2.20)	2.70 (1.00-7.70)	1.8 (1.7,2.0)
South Asia	11089.30 (7019.40-16221.70)	16661.40 (6747.90-46913.90)	0.50 (-0.58, 5.68)	2.60 (1.60-3.80)	3.20 (1.30-9.10)	0.6 (0.4,0.7)
Southeast Asia	2996.00 (1910.90-4338.50)	5225.60 (2096.30-15897.00)	0.74 (-0.52, 7.32)	1.70 (1.10-2.50)	3.10 (1.20-9.30)	1.8 (1.6,1.9)
Western Europe	7678.50 (6265.30-9460.30)	15918.00 (8431.70-36020.00)	1.07 (-0.11, 4.75)	10.80 (8.80-13.30)	23.20 (12.30-52.60)	2.2 (2.0,2.5)
Western Sub-Saharan Africa	451.40 (275.10-732.60)	1645.90 (616.10-4339.70)	2.65 (-0.16, 14.77)	0.50 (0.30-0.80)	0.70 (0.30-1.90)	1.1 (1.0,1.2)
