# Supplementary material for: Diverging regional inequalities in childhood diabetes, 1990–2023: a GBD 2023-based analysis
Source: Pediatr Diabetes. 2026 Jun 11;2026:e001. doi: 10.48130/pedi-0026-0002 (PMC13463378; doi:10.48130/pedi-0026-0002)
Supplement: Supplementary file 1 [file pedi-2026-2026-1-0002-S1.zip › 10.48130_pedi-0026-0002-Suppl-TableS2.pdf]

Appendix Table 2. Epidemiological Patterns of Childhood Diabetes Mortality Across GBD Regions, 1990 – 2023

Location	Deaths 1990 (Number)	Deaths 2023 (Number)	Number % Change	Deaths 1990 (Rate)	Deaths 2023 (Rate)	EAPC (95% CI)
Australasia	2.40 (2.10-2.70)	1.50 (1.30-1.70)	-0.38 (-0.52, -0.19)	0.10 (0.00-0.10)	0.00 (0.00-0.00)	-2.8 (-3.1,-2.5)
Central Asia	54.30 (47.20-66.00)	71.40 (60.90-84.50)	0.31 (-0.08, 0.79)	0.20 (0.20-0.30)	0.20 (0.20-0.30)	0.1 (-0.2,0.4)
Central Europe	23.80 (20.80-28.60)	4.90 (4.20-5.50)	-0.79 (-0.85, -0.74)	0.10 (0.10-0.10)	0.00 (0.00-0.00)	-3.0 (-3.2,-2.8)
Central Sub-Saharan Africa	290.00 (169.10-430.20)	349.60 (200.50-523.40)	0.21 (-0.53, 2.10)	1.10 (0.70-1.70)	0.60 (0.30-0.80)	-2.0 (-2.1,-1.8)
East Asia	616.30 (362.50-933.60)	101.80 (62.90-151.60)	-0.83 (-0.93, -0.58)	0.20 (0.10-0.30)	0.00 (0.00-0.10)	-4.8 (-5.0,-4.6)
Eastern Europe	69.40 (62.50-75.60)	36.40 (32.00-41.00)	-0.48 (-0.58, -0.34)	0.10 (0.10-0.10)	0.10 (0.10-0.10)	-0.7 (-1.6,0.2)
Eastern Sub-Saharan Africa	1440.00 (865.90-2210.30)	1121.30 (681.80-1605.70)	-0.22 (-0.69, 0.85)	1.60 (1.00-2.50)	0.60 (0.40-0.90)	-3.1 (-3.2,-3.0)
High-income North America	55.80 (49.90-63.50)	47.80 (42.00-54.50)	-0.14 (-0.34, 0.09)	0.10 (0.10-0.10)	0.10 (0.10-0.10)	-1.3 (-1.7,-1.0)
North Africa and Middle East	1095.90 (650.20-1973.10)	577.00 (362.00-985.50)	-0.47 (-0.82, 0.52)	0.80 (0.50-1.40)	0.30 (0.20-0.50)	-2.4 (-2.6,-2.3)
Oceania	32.10 (17.90-51.20)	43.30 (25.80-63.90)	0.35 (-0.50, 2.57)	1.20 (0.70-1.90)	0.80 (0.50-1.20)	-1.2 (-1.4,-1.0)
South Asia	1271.50 (796.30-2216.30)	584.10 (326.60-821.20)	-0.54 (-0.85, 0.03)	0.30 (0.20-0.50)	0.10 (0.10-0.20)	-3.0 (-3.1,-2.9)
Southeast Asia	497.00 (280.70-740.90)	244.40 (123.90-371.60)	-0.51 (-0.83, 0.32)	0.30 (0.20-0.40)	0.10 (0.10-0.20)	-2.2 (-2.3,-2.1)
Western Europe	57.80 (51.50-65.20)	16.50 (14.50-18.50)	-0.71 (-0.78, -0.64)	0.10 (0.10-0.10)	0.00 (0.00-0.00)	-4.0 (-4.1,-3.8)
Western Sub-Saharan Africa	1708.90 (656.00-2909.70)	2201.70 (844.60-3669.70)	0.29 (-0.71, 4.59)	1.90 (0.70-3.30)	1.00 (0.40-1.60)	-2.1 (-2.2,-2.0)
