# Supplementary material for: Diverging regional inequalities in childhood diabetes, 1990–2023: a GBD 2023-based analysis
Source: Pediatr Diabetes. 2026 Jun 11;2026:e001. doi: 10.48130/pedi-0026-0002 (PMC13463378; doi:10.48130/pedi-0026-0002)
Supplement: Supplementary file 1 [file pedi-2026-2026-1-0002-S1.zip › 10.48130_pedi-0026-0002-Suppl-TableS3.pdf]

Appendix Table 3. Epidemiological Patterns of Childhood Diabetes DALYs Across GBD Regions, 1990–2023

Location	DALYs (Disability-Adjusted Life Years) 1990 (Number)	DALYs (Disability-Adjusted Life Years) 2023 (Number)	Number % Change	DALYs (Disability-Adjusted Life Years) 1990 (Rate)	DALYs (Disability-Adjusted Life Years) 2023 (Rate)	EAPC (95% CI)
East Asia	53443.30 (31885.80-80065.30)	10744.30 (7128.10-14993.00)	-0.80 (-0.91, -0.53)	16.20 (9.70-24.30)	4.10 (2.70-5.80)	-4.2 (-4.4,-4.1)
Eastern Sub-Saharan Africa	123616.10 (74585.30-189980.20)	96703.70 (59730.20-137684.00)	-0.22 (-0.69, 0.85)	137.80 (83.10-211.80)	51.70 (32.00-73.70)	-3.1 (-3.1,-3.0)
South Asia	106807.90 (68458.60-185649.10)	51800.70 (30041.90-70180.10)	-0.52 (-0.84, 0.03)	25.10 (16.10-43.60)	10.00 (5.80-13.60)	-2.8 (-2.9,-2.7)
North Africa and Middle East	94331.20 (56220.40-167871.10)	55076.30 (37386.40-88694.90)	-0.42 (-0.78, 0.58)	66.70 (39.80-118.70)	29.90 (20.30-48.20)	-2.1 (-2.3,-2.0)
Southeast Asia	41751.60 (23865.50-61498.10)	21316.00 (11472.60-32065.60)	-0.49 (-0.81, 0.34)	24.40 (13.90-35.90)	12.50 (6.70-18.70)	-2.1 (-2.2,-2.0)
Western Sub-Saharan Africa	145443.50 (55291.60-248035.40)	187187.70 (71992.80-313001.00)	0.29 (-0.71, 4.66)	164.70 (62.60-280.80)	83.60 (32.10-139.80)	-2.1 (-2.2,-2.0)
Central Sub-Saharan Africa	24406.30 (14266.80-36252.10)	29275.20 (17026.80-43593.80)	0.20 (-0.53, 2.06)	95.60 (55.90-142.10)	46.30 (27.00-69.00)	-2.0 (-2.1,-1.9)
High-income North America	7234.90 (5892.70-8687.30)	11860.30 (8349.00-17472.40)	0.64 (-0.04, 1.97)	11.70 (9.50-14.10)	18.00 (12.70-26.50)	1.3 (1.1,1.4)
Oceania	2710.90 (1528.00-4298.30)	3650.30 (2187.60-5375.20)	0.35 (-0.49, 2.52)	99.90 (56.30-158.40)	67.20 (40.30-99.00)	-1.2 (-1.4,-1.0)
Central Europe	2479.30 (2104.60-3060.20)	1770.70 (1172.40-2727.50)	-0.29 (-0.62, 0.30)	8.30 (7.00-10.20)	10.00 (6.60-15.40)	1.0 (0.7,1.3)
Eastern Europe	6709.70 (5845.80-7704.90)	3876.00 (3131.00-4804.40)	-0.42 (-0.59, -0.18)	13.00 (11.30-14.90)	12.30 (9.90-15.30)	-0.4 (-1.1,0.3)
Western Europe	6998.00 (5942.60-8182.90)	5935.10 (3834.90-9191.40)	-0.15 (-0.53, 0.55)	9.80 (8.40-11.50)	8.70 (5.60-13.40)	-0.3 (-0.4,-0.1)
Central Asia	4647.60 (4074.90-5506.80)	6253.40 (5284.50-7402.70)	0.35 (-0.04, 0.82)	18.40 (16.10-21.80)	21.10 (17.90-25.00)	0.2 (-0.0,0.5)
Australasia	356.10 (288.00-445.70)	488.40 (333.70-693.40)	0.37 (-0.25, 1.41)	7.70 (6.30-9.70)	8.40 (5.70-11.90)	-0.1 (-0.3,0.1)
